# Healthcare access for children in a low-income area in Cape Town: A mixed-methods case study

**DOI:** 10.4102/safp.v65i1.5754

**Published:** 2023-12-20

**Authors:** Luke B. Profitt, Graham Bresick, Liezel Rossouw, Ben van Stormbroek, Tasleem Ras, Klaus von Pressentin

**Affiliations:** 1 Department of Family Medicine, Faculty of Health Sciences, University of Cape Town, Cape Town, South Africa; 2 Western Cape Government Health and Wellness, Cape Town, South Africa; 3 Western Cape Government Health and Wellness, False Bay Hospital, Cape Town, South Africa; 4 Western Cape Government Health and Wellness, Victoria Hospital, Wynberg, Cape Town South Africa; 5 Department of Paediatrics, Faculty of Health Sciences, University of Cape Town, Cape Town, South Africa

**Keywords:** barriers, facilitators, healthcare access, children, low-income area

## Abstract

**Background:**

In Cape Town, the under-5 mortality rate has plateaued to 20 per 1000 live births, with 60% of child deaths occurring out of hospital. The southern subdistrict has the largest paediatric population in Metro West and accounts for 31% of deaths. This study aimed to uncover the access barriers and facilitators underlying this high burden of out-of-hospital deaths.

**Methods:**

An exploratory mixed-methods case study design employed three data collection strategies: a quantitative survey with randomly sampled community members, semi-structured interviews with purposively sampled caregivers whose children presented critically ill or deceased (January 2017 – December 2020) and a nominal group technique (NGT) to build solution-oriented consensus among purposively sampled health workers, representing different levels of care in the local health system.

**Results:**

A total of 62 community members were surveyed, 11 semi-structured caregiver interviews were conducted, and 11 health workers participated in the NGT. Community members (74%) experienced barriers in accessing care. Knowledge of basic home care for common conditions was limited. Thematic analysis of interviews showed affordability, acceptability, and access, household and facility factor barriers. The NGT suggested improvement in community-based services, transport access and lengthening service hours would facilitate access.

**Conclusion:**

While multiple barriers to accessing care were identified, facilitators addressing these barriers were explored. Healthcare planners should examine the barriers within their geographic areas of responsibility to reduce child deaths.

**Contribution:**

This study uncovers community perspectives on childhood out-of-hospital deaths and makes consensus-based recommendations for improvement.

## Introduction

The poorest and the most rural communities are usually the worst affected by high childhood mortality rates, usually because of barriers to accessing care.^1,2,3^ This is demonstrated (2021 World Bank figures) in the differences in under-5 mortality rates (U5MR) between high-income countries (e.g., Spain) with 5 per 1000 live births and sub-Saharan Africa with 76 per 1000 (50% of the global child deaths).^2,4^ In response to the poverty-related burden of illness, the United Nations published Millennium Development Goals (MDGs).^5^ The fourth MDG was to reduce deaths under 5 years by two-thirds. Globally, there has been only a 50% drop in under-5 mortality.^5^ The MDGs were replaced in 2016 with the Sustainable Development Goals (SDGs),^5^ following the 2015 report that noticed the interplay of poverty and health, with vulnerable children living in poverty suffering double the mortality compared with those from the richest quintiles of society.^5^ The SDGs attempt to address the Social Determinants of Health (SDH) that reflect the circumstances in which people live such as education levels, sanitation and nutrition.^1,5,6^

Barriers to accessing healthcare are common^7^ in developing countries and vulnerable populations across the globe and have been described in terms of availability, affordability and accessibility. Accessibility includes access to transportation and communication in one’s own language.^8,9^ Availability of healthcare facilities tends to vary between urban and rural settings, with 62% of African people having a clinic within walking distance.^7^ Affordability relates to both the direct cost such as user fees and medication costs and the indirect costs such as loss of income or transport costs.

South Africa has reduced U5MR from 75 per 1000 in 2008 to 34 per 1000 in 2016. This varies by province and population group reflecting the apartheid segregation and the rural-urban divide.^2,10^ The Western Cape province U5MR plateaued in 2018 at 20 per 1000 live births^11^ from 24.1 in 2011.^11^ By 2015, the City of Cape Town had succeeded in a 33% drop in the U5MR to 19 per 1000.^12,13,14^ The reduction is because of improved HIV care and reduction of diarrhoeal deaths.

The plateauing of the death rate has been linked to out-of-hospital preventable deaths making up 50% – 70% of all under-5 deaths, similar to the rest of Southern Africa where 50% of deaths are out of the hospital,^4^ from pneumonia, gastroenteritis, trauma and sepsis.^9,12,14,15^ Pathway to care^16^ studies have shown delays in seeking care are because of caregivers’ failure to recognise illness or severity thereof, lack of transport, healthcare facility issues and other barriers.^4,17^ A retrospective study of under five deaths in Metro West in 2011 showed that up to 15% of children dying at home had received care within the week that they died.^14^ In Cape Town, there are complex pathways to access care with multiple delays from appropriate health-seeking behaviour, assessment and transfer to a Paediatric Intensive Care Unit (PICU) resulting in potentially avoidable deaths.^18^ Common barriers to care within Cape Town include language communication challenges and facility barriers, including staff attitudes and access issues.^19^ Most patients attending the tertiary referral centre, Red Cross War Memorial Children’s Hospital (RCWMCH), are walking (70%) to access their closest healthcare with a median distance to a 24-h facility 6 km, with some up to 13 km from their home.^18,19^

The key research questions this study aimed to answer were, concerning the U5MR, what are the perceived barriers to care and how can access be facilitated? The outcomes of interest include reviewing health-seeking behaviour, understanding the access to care experiences of caregivers including identifying barriers to care, and building consensus around proposed solutions to addressing these barriers. There are no studies in our setting that explore both barriers to accessing care and propose solutions. The current study aims to bridge this gap by not only amplifying the voices of those who have experienced barriers to accessing healthcare for children but also bringing a consensus of possible solutions from the full spectrum of healthcare workers immersed in the context, from community health workers (CHWs) to the outreach paediatrician (specialist from referral centre).

## Research methods and design

### Study design

This was an exploratory case study mixed-methods design with a convergent parallel approach.^20^ The quantitative component employed survey methodology, run parallel to the qualitative arm where semi-structured interviews were conducted. The data generated by these components converged to inform deliberations in the Nominal Group Technique (NGT). The NGT is a consensus-seeking methodology designed to generate a list of prioritised items about a particular phenomenon, in this instance, out-of-hospital child deaths.^21^

### Setting

The southern subdistrict, one of eight in the City of Cape Town, has a large paediatric population and the largest U5MR, and out-of-hospital mortality. The ‘far south’ is a sub-area within the southern subdistrict and is served by False Bay Hospital (FBH), a family physician-led district healthcare facility with 76 inpatient beds, of which six are paediatric beds. The 24-h emergency centre (EC) has five assessment beds and six overnight beds that are shared between adult and paediatric patients depending on demand. These patients are either self-referred or referred in from the surrounding clinics. Common paediatric emergencies include gastroenteritis with shock, pneumonia or bronchiolitis with severe respiratory distress, status epilepticus and acute exacerbations of asthma.

Community health workers, employed by non-governmental organisations (NGOs), provide services within the community and home settings. The community has 75% informal housing and is home to many migrants and others who were marginalised historically. Three primary health care (PHC) facilities provide an 8-h weekday service with one satellite site. The community constitutes diverse ethnic groups, predominantly Afrikaans-speaking mixed-race, isiXhosa-speaking indigenes, as well as migrant Africans from various countries. Seventy-five percent of these people reside in informal housing. In addition, high unemployment, crime and low educational levels are significant social determinants of health (verbal report from CHWs).

### Study population and sampling strategy

For the community-based survey, the study population was those adults (18 years and older), living in Masiphumelele, with direct responsibility for providing care to a child (termed caregivers throughout the rest of the study), and able to communicate (written or verbal) in English, Afrikaans, isiXhosa, Chichewa or Shona. Cluster sampling was performed in each of the four quadrants in formal housing areas, government housing and informal areas seeking out also foreign nationals to get a balanced representation of Masiphumelele using a map drawn by a local NGO, Living Hope. Adults who were not caregivers or could not communicate in any of the five languages were excluded.

The sample size was calculated at 62, using Calculator.net sample calculator,^22^ to obtain a representative response from the population of 40 000 representing 10 000 households with an expected rate of 80% experiencing barriers, a margin of error (precision) of 10% and a confidence interval of 95%.

Inclusion criteria for the semi-structured interviews were caregivers. This term is defined as people who care for children and includes parents or others, who brought children to FBH who were coded either red (extremely ill and requiring immediate emergency care) or blue (dead on arrival). Caregivers were excluded if they were under 18 years of age, unable to communicate in South African languages dominant in the Western Cape (isiXhosa, Afrikaans or English), contact details were unavailable; were unable to attend the interview and those who were incorrectly triaged into a higher acuity level. The additional files found were returned from the mortuary and added to the database.

For the consensus building exercise using the NGT,^21^ 11 participants were purposively recruited from the health services to ensure representation: five CHWs, one family physician, one clinic nurse, two nurse supervisors for CHWs, the outreach paediatrician and the head of the local NGO. This group already forms an existing community orientated primary care (COPC) structure known as the Far South Clinical Governance Forum. Emergency medical services (EMS) are also usually represented in the group but were excluded, unfortunately, as they were not available on the day.

### Data collection

Data collection tools included a community survey questionnaire, semi-structured interviews with caregivers and a NGT-based meeting with healthcare workers.

A questionnaire (see Online Appendix 1) was developed based on the World Health Organization (WHO) verbal autopsy^23^ to interview parents and caregivers in the community. The WHO verbal autopsy tool is used to postulate the cause of death and possible reversible causes in areas where there is limited access to a formal mortuary and diagnostic services. An initial pilot of the questionnaire was carried out with five respondents; feedback was obtained on clarity regarding content and constructs, and the survey was adapted accordingly. Furthermore, feedback was obtained from the translators and amendments were made. The survey translations – Afrikaans, isiXhosa and Shona – were checked by a second native speaker for accuracy. The questionnaire was administered by a research assistant in paper-based format or self-administered in electronic format (Google Forms) between April 2020 and September 2020.

Semi-structured interviews were conducted with caregivers whose children died or survived a major critical health event. Interviews were conducted by an independent, trained research assistant, who is a professional counsellor, and recorded on digital devices between November 2020 and May 2021. The semi-structured interviews (see Online Appendix 2) explored demographic information, and the experience of caregivers in accessing care, including language fluency, physical barriers and social factors associated with access to healthcare. A translator was used during the interview for non-English language speakers, while confidentiality was ensured. Recordings were transcribed verbatim by a separate assistant. The primary researcher checked the transcriptions for accuracy and anonymity by comparing audio recordings with transcripts. Interviewees experiencing grief reactions were offered voluntary counselling with a local NGO (HospiVision).

The NGT participants were tasked with answering the question ‘How can we improve access to healthcare for children in the Far South?’ after being presented with the preliminary findings of the survey and interviews at a physical meeting. As per NGT, participants were asked to collectively generate a list of suggestions and then vote on their preferred options using a feasibility-importance framework to rank both importance of the intervention and how feasible it would be to implement. Following the silent phase of recording their ideas, there was a process of item generation and clarification. The voting and prioritisation were performed electronically and scored to generate the consensus.

### Data analysis

The survey data were analysed using basic spreadsheet tools. The responses to open-ended questions were analysed using the framework analysis.^24^ The interview data were analysed by manually coding key phrases from the transcriptions using NVIVO12 for windows (2021, QSR International) and confirmed independently by two other researchers to determine themes from within the narratives of the interviews. No new interviews were conducted once theme saturation had been reached and no new data emerged. All the data collected were de-identified and stored in a password-protected computer file.

### Ethical considerations

Ethics approval was obtained from the University of Cape Town (UCT) Health Research Ethics Committee (Reference: HREC 869_2019) and permission was obtained from Western Cape Government Health and Wellness (WCGHW) (Reference: WC_202003_020) and the City of Cape Town (Reference: 8306) to conduct this research study. Participation in all aspects of the study was voluntary and informed consent was obtained. Data for each of the three methods were anonymised and stored securely after being captured and electronic data were stored in password-protected electronic storage, confidentiality, risks and benefits were explained.

## Results

Sixty-two community members were surveyed, 11 caregiver interviews were conducted, and 11 health workers participated in the NGT. The following subsections will unpack the findings from each method, before highlighting how the NGT assisted with integrating the findings as part of the mixed-methods design.

### Community survey

Table 1a and b shows the community’s demographics obtained through the survey and insight into health-seeking behaviour patterns.

**TABLE 1a:** Results of the community survey.

Variable	Category	*n*	%
**Demographic information for respondents (*N* = 62)**			
Gender of participant	Female	48	77
	Male	9	14
	Undisclosed	5	8
Nationality	South African	47	76
	Zimbabwean	10	16
	Malawian	5	8
Housing	Brick	18	29
	Wendy (informal wooden housing)	4	6
	Shack (wood, plastic and iron sheeting)	38	61
Schooling completed	Never attended	3	5
	Primary	11	18
	Secondary	40	65
	Tertiary	6	10
	Other	1	2
Home language	isiXhosa	39	63
	Shona	7	11
	Chichewa	3	5
	Afrikaans	3	5
	English	1	2
Languages used in the health centre to communicate with patients	IsiXhosa	18	29
	English	17	27
	Mixed English and IsiXhosa	22	35
	Other	5	8
Frequency of health care visits	Seldom (< 2 visits per year)	15	24
	Infrequent (2–4 visits per year)	40	65
	Regularly (5–6 visits per year)	11	18
	Frequently (> 7 visits per year)	9	14
**Perception of access to care**			
Experienced difficulty in accessing care for children	-	45	74
Barriers experienced	Long waiting times	49	79
	Staff attitude	17	27
	Transport	8	13
	Language	7	11
	Knowledge of system	5	8
	Fears	5	8
	Cost	4	6
**I feel that health services are accessible**		55	89
Mode of transport to the healthcare facility	Walking	21	34
	Neighbour’s car	11	18
	Own transport	7	11
	Ambulance	8	13
	Public transport	11	18
**Correct Knowledge of emergency services number**		36	60
I feel the staff’s attitude towards me is *(followed by the relevant descriptor in the options listed)*	Good	6	10
	Caring	10	16
	Abrupt	1	1
	Too busy	15	24
	Helpful	5	8
	Bad	1	1
	Good and caring	1	1
	Kind	17	27
	Did not answer	6	10
I believe the health system (ambulance, clinic, hospital) is *(followed by the relevant descriptor in the options listed)*	Poor	20	32
	Moderate	6	10
	Good	4	6
	Excellent	22	35
Reason for descriptor selected	I heard so	9	18
	My experience	35	56
	I don’t know	18	29
	They can’t speak my language	5	8
**Health seeking behaviour diarrhoea**			
When your child becomes ill with diarrhoea, what should you do?	Initiate sugar salt solution at home	20	32
	Take to the clinic first	25	40
	Take the child straight to the hospital	11	18
	Give traditional medication	1	2
When your child is sick with diarrhoea *(followed by the relevant descriptor in the options listed)*	Restrict food and water	2	3
	Give sugar salt solution	22	35
	Take the child to the clinic	21	33
	Take the child to the hospital	10	16
During illness would you take your child to the clinic or the hospital *(followed by the relevant descriptor in the options listed)*	Immediately	16	25
	When having loose stool and thirst	25	40
	When becoming weak	8	12
	When too weak to take sugar salt solution	9	14
If a child in your care is sick with runny stools (diarrhoea) I would take them to (*followed by the relevant descriptor in the options listed*)	Clinic	41	66
	Use home or plant remedies	1	2
	Take the child to hospital	12	19
	Give sugar salt solution	5	8
**Vomiting**			
If a child in your care is sick with vomiting, I would take them to *(followed by the relevant descriptor in the options listed)*	Clinic	47	75
	Take to spiritual healer or spiritist to drive out spirits afflicting them	4	6
	Take the child to hospital	5	8
	Give sugar salt solution	2	3
**Cough with fever**			
If a child is coughing with a fever, when do you need to take them to the hospital or clinic?	Within hours	11	18
	During cough and fever	11	18
	When looks ill	10	16
	If not improving after 1 day	18	29
	If not improving after 2 days	5	8
	If struggling to breathe	2	3
If a child in your care is sick with a cough with fever, I would take them to *(followed by the relevant descriptor in the options listed)*	Clinic	43	69
	Private GP	1	1
	Pray for them	7	11
	Use over the counter medicines	1	1
	Take the child to hospital	9	14
	Give sugar salt solution	2	3
**Injury**			
I would take my child to the hospital (Selection of *the relevant option of Wong-Baker pain faces*)†	No hurt	2	3
	Hurts a little bit	11	18
	Hurts even more	0	0
	Hurts a whole lot	11	18
	Hurts worst	4	6

GP, general practitioner.

† , In total, 91% of respondents would take their child to the hospital if there was any level of pain following trauma. This was clarified further with the following question based on the Wong-Baker pain faces.^25^

**TABLE 1b:** Results of the community survey.

Fluency in local languages	High fluency		Moderate fluency		Poor fluency		Very poor fluency	
	*n*	%	*n*	%	*n*	%	*n*	%
English	28	45	16	26	15	24	3	4
Afrikaans	5	8	8	13	14	23	33	53
isiXhosa	45	73	4	6	7	11	6	9

While most participants feel the services are accessible, many experience barriers in accessing them, with more than a quarter (27%) experiencing multiple barriers to access. The results on perceptions of staff attitudes are divergent with more respondents selecting positive attitudes such as care and kindness but a significant number (24%) selecting that they are too busy. Participant demographics indicate a multicultural community, high unemployment and mostly inadequate housing. Almost one-quarter of survey respondents were foreign nationals who experienced additional barriers to accessing services including language, poor attitudes of healthcare workers and expensive after-hour transport.

### Qualitative section of survey

The open-ended section (see Online Appendix 1) of the survey showed overall poor health-seeking behaviour and knowledge of home treatment of basic conditions. Themes were grouped into a framework that had been determined a priori, namely accessibility, affordability, acceptability, household factors and facility factors. Some of the community assets recognised by survey respondents to overcome these barriers included the unity of the community and care for the environment and education.

### Caregiver interviews

**FIGURE 1:**
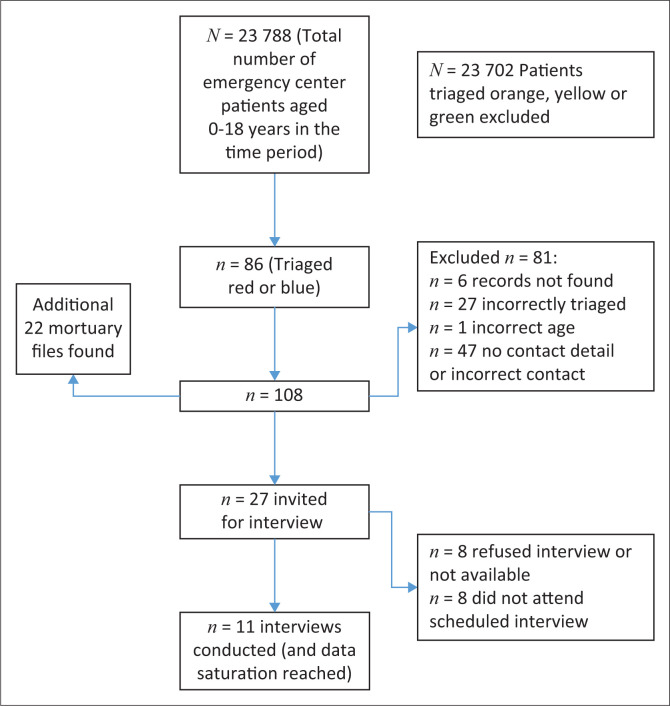
Flow diagram of interview participants.

Eleven primary caregivers were interviewed. Figure 1 shows recruitment and selection of interview candidates. Table 2 shows the demographic data and EC dispositions of the children whose caregivers participated in the interviews. Framework data analysis^24^ grouped the themes into six categories – affordability, acceptability, accessibility, availability, knowledge and understanding/household factors and facility factors – with associated subcategories. As expected, there was an overlap between accessibility, acceptability, availability and facility factors as barriers to care.

**TABLE 2:** Demographic data of the children of interview participants.

Participant	Interviewee relationship to child	Age of child at presentation	Home language (nationality)	Diagnosis of child	EC disposition
A1	Mother	11 years 9 months	Xhosa (South African)	Meningitis	Transferred to specialist paediatric service, admitted to ICU
A2	Mother	15 years 10 months	Xhosa (South African)	Epilepsy -– breakthrough seizures	Admitted to FBH
A4	Father	2 years 7 months	Nyakyusa (Malawian)	Acute severe gastroenteritis	Admitted to FBH
A28	Mother	6 months	Shona/English (Zimbabwean)	Broncho-pneumonia	Discharged home
A32	Mother	1 year 6 months	English (South African)	Pharyngitis	Discharged home
A33	Mother	2 years 1 month	Shona (Zimbabwean)	Chickenpox	Discharged home
A39	Mother	1 year 7 months	English (South African)	Febrile convulsion likely because of viral infection	Admitted to FBH
A58	Mother	2 months 21 days	Afrikaans (South African)	Possible SIDS versus sepsis	Declared dead in EC
A59	Father	7 years 5 months	English (South African)	Gunshot chest	Dead on arrival
A65	Mother	1 month 24 days	English and Afrikaans (South African)	Sepsis, pneumonia	Dead on arrival
A66	Mother	1 year 1 month	Chichewa (Malawian)	Poison/cleaning substance ingestion	Admitted overnight for observation

EC, emergency centre; FBH, False Bay Hospital; ICU, intensive care unit; SIDS, Sudden Infant Death Syndrome.

A biological parent was the primary caregiver in all instances, with mothers making up most interviewees (*n* = 9/11). The first language included isiXhosa (*n* = 2/11), English (*n* = 4/11), Afrikaans (*n* = 1/11) and foreign African language (*n* = 4/11). One of the respondent’s children was declared dead on arrival, and one was admitted to a tertiary-level intensive care unit, while the balance was either treated at the district hospital or discharged home. The diagnoses include pneumonia, gastroenteritis, meningitis, trauma and poisoning. Four children were under 1 year, five were between 1 year and 5 years, and two were over 5 years. Table 2 shows the diagnosis and EC disposition of the children.

The cost of accessing care: The loss of income and potentially the loss of employment for being absent makes accessing healthcare unaffordable for some. This financial barrier is indicated clearly in the following quotes:

‘I will not stay because I am breadwinner in this house’ … once I take off day. yho!’ (Female, English, survey respondent 20)

‘Not enough money for the taxi sometimes …’ (Female, Shona, interview respondent A28)

‘… [*Y*]ou need help you get even more frustrated because you specifically set up that day [*and not able to earn*].’ (Male, English, interview respondent A59)

The physical location or geographic accessibility of the health service is influenced by distance and transport availability where the experience of people is that the ambulance would not come to them, with Chichewa survey respondents reporting after-hour transport costs of R350.00 – R500.00. Safety issues pose a concern and prevent access. Access is further hindered by the language spoken by the healthcare staff. The following quotes depict this:

‘I am working and I don’t have a car to carry my family to the hospital if they fall sick especially during the night.’ (Male Chichewa, survey respondent 28)

‘I will not stay because I don’t understand the English they use in hospital.’ (Female, Shona, survey respondent 39)

‘Makhula. It’s ah a little bit far.’ (Female, Xhosa, interview respondent A1)

‘… [*W*]e just called Uber to come and get him to the hospital … I didn’t think about [*calling ambulance*] because he … it take long …’ (Female, Chichewa, interview respondent A66)

‘For safety reasons because the area that the clinic is in, there’s always gangsters around.’ (Male, English, interview respondent A59)

Respondents engaged with ‘acceptability’ by discussing the perceived work ethic, attitude and training of staff. A sense of not being cared for came through quite strongly in the following quotes:

‘ … [*Y*]ou can’t speak to them because they are irritated … it’s almost like they are trying to show me, they tried to do something but they are not … Three/four doctors they didn’t say much, they just said sorry until I had to go and get something to calm myself.’ (Female, Afrikaans, interview respondent A58)

‘… [*A*]sked the security to help … because the child, … was getting dizzy but the security ignored [*us*].’ (Female, Xhosa, interview respondent A1)

‘… [*Y*]ou almost died, and they didn’t care about it.’ (Male, Nyakyusa, interview respondent A4)

‘I think they’re not trained well enough to engage themselves into the lives of people that come …’ (Female, English, interview respondent A65)

However, these experiences are challenged by a single comment from a foreigner, who had positive comments about staff:

‘Everything is perfect, I know, and I tell you as a foreigner we experience no different, some minorities but in False Bay, I tell you maybe the whole Cape Town there is no number one hospital … but False Bay they treat the same, no different to anybody.’ (Male, Nyakyusa, interview respondent A4)

Respondents reported that they made the decision not to access care, citing long waiting times as a key barrier. However, they did not report what alternative options they put in place:

‘Well, it’s like, if you come in for emergencies, like, you wait forever.’ (Female, Afrikaans, interview respondent A58)

‘… [*S*]ometimes people are turned away because they just can’t. They don’t have the capacity to deal with you today …’ (Female, English, interview respondent A65)

‘I leave my problem that I have because I see other people with more serious problems come in hospital …’ (Female, Chichewa, interview respondent A66)

‘… [*T*]hat they maybe just offer the child a snack … There would be parents sitting … They think they are just going to see a doctor and then go home so they don’t pack a snack.’ (Female, English, interview respondent A32)

Knowledge of illness, safe home remedies and ways of accessing emergency care emerged as key issues. These findings suggest a low sense of self-efficacy in caring for severely ill children:

‘… [*H*]is daddy stopped him and uh he take some soap and he cleaned the mouth …’ [*home treatment for tox in ingestion*] (Female, Chichewa, interview respondent A66)

‘When I want to come to hospital … They said you must go to clinic first and then one will send you here. It’s just their rules, I don’t know why.’ (Female, Shona, interview respondent A33)

‘… [*I*]t’s interesting we all know the nine-one-one for America, but we don’t know our own one …’ (female, English, interview respondent A65)

‘… [*B*]ecause most of the time he is with my mother because I work a lot so …’ (Female, English, interview respondent A39)

‘… I’ve got one lady who I am working for. Sometimes he help me a lot …’ (Female, Shona, interview respondent A33)

### Characteristics of paediatric patients at False Bay Hospital

**FIGURE 2:**
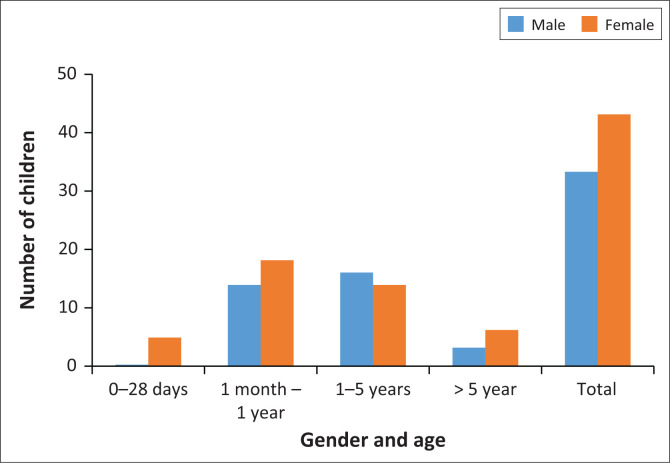
Distribution of paediatric emergency visits to False Bay Hospital by age and gender.

**FIGURE 3:**
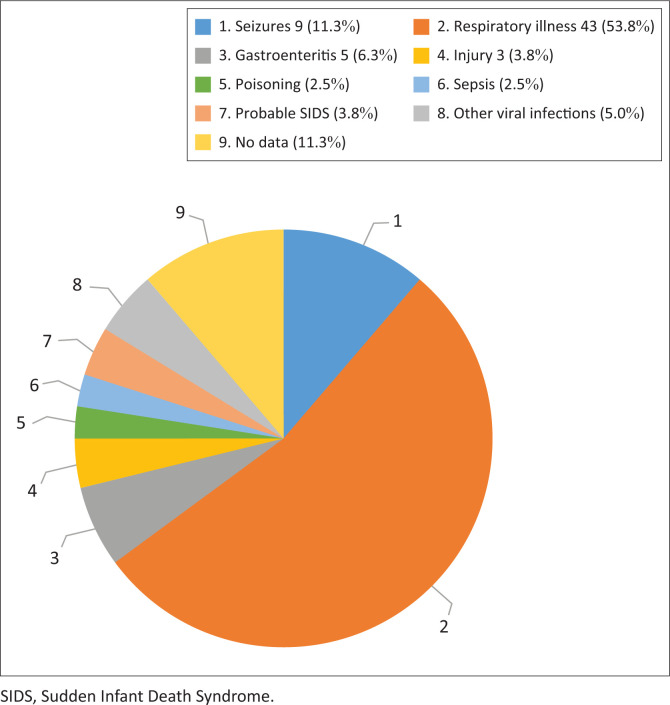
Diagnoses or presenting complaint.

False Bay Hospital records indicate that 23 788 children attended the EC between January 2017 and December 2020, giving an average of 5947 per year. Of these, 86 were triaged red or blue code according to the SATS. This is an average of 21 (0.35%) per year or just under two patients per month. A further 22 records (files) were found (returned from the mortuary to the hospital) that did not appear in the EC records (see Figure 1). The age category 1 month to 1 year was the largest, with a female predominance – female children predominated in all age categories except 1–5 years (see Figure 2). The most common presenting complaint or diagnoses were respiratory illnesses (53.8%), seizures (11.3%), gastrointestinal conditions (6.3%), sepsis including neonatal sepsis (2.5%), presumed Sudden Infant Death Syndrome (SIDS) (3.8%), injuries (3.8%) and poison ingestion (2.5%), (see Figure 3). The EC dispositions showed that 15 children transferred to higher levels of care (Level 2 or Level 3), 20 admitted at the district level (Level 1 – generalist care), five were declared dead in the EC and five were dead on arrival, and there were insufficient data in four cases to confirm if they were in hospital deaths or not. Four absconded before being seen, 13 had no record of their outcome and 42 were discharged home.

In cases where cause of death was uncertain if natural or unnatural or in the cases of definite unnatural causes autopsy was requested as per departmental protocol. It was not within the scope of this study to correlate the cause of death recorded in the file with the findings of the forensic pathology services.

### Nominal group technique engagement

**FIGURE 4:**
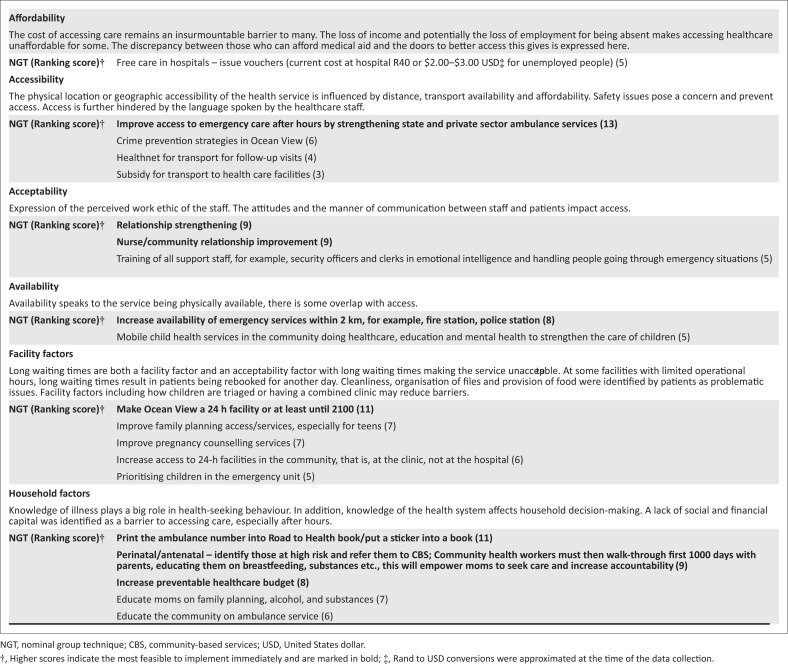
Health worker proposed solutions and ranking using the nominal group technique.

The results of the nominal group process that generated and ranked solutions to community-identified problems are presented in Figure 4. The solutions in bold were found to have high levels of consensus among respondents. In the NGT, participants ranked solutions according to those that would be most helpful and practical to implement.

## Discussion

This exploratory mixed-methods design study aimed to determine barriers to healthcare access for children in the southern subdistrict (or ‘Far South’) of the City of Cape Town and to identify facilitators directed at reducing paediatric mortality. A survey of caregivers of children and interviews with community members were utilised to collect qualitative and quantitative data. These data were presented to the NGT consisting of a range of healthcare workers immersed in this context to obtain consensus on how to address these barriers. The NGT consensus supported the principal investigator’s (L.B.P.) hypothesis that many deaths may be related to health seeking behaviours as well as barriers to accessing care.

### Health seeking behaviour

Despite most respondents indicating a preference for Western medicine over traditional medicine, our findings show that basic knowledge of healthcare for children at home is poor along with poor health-seeking behaviour. Most caregivers did not know when to give oral rehydration, which conditions could be managed at home, or when to seek care at a health facility. Only a third of survey participants would use the WHO standard of sugar salt solution (SSS)^26^ to treat diarrhoea. This reflects poor insight into home-based care for children with gastroenteritis, a common condition accounting for 10% of childhood deaths in the Metro West area of Cape Town.^14^

Every child born in South Africa is given a Road to Health Booklet (RTHB) which details what to do and where to go in the event of a sick child. The study highlights that these guidelines are not followed, defying one of the key principles of the first 1000-day programme.^27^ In addition, recognition of the severity of illness is poor. Some caregivers either rush to take a child in their care to the clinic at the first signs of a cough with a fever or immediately the child has diarrhoea or seek help too late. There is no indication in the data or studies that aided our understanding of these extremes in health-seeking behaviour. Poor recognition of illness severity is a global phenomenon.^28,29^ Too many presentations of mild illnesses have the potential to overburden services while delayed presentations can have morbidity or mortality impacts; however, recognition of the severity of the illness can, however, be difficult even for healthcare providers.^18^ Health education of parents is likely to assist with the moderation of this behaviour.

An interesting finding is that there were very few neonates (under 28 days) presenting to the hospital’s EC during the study period. There were similar numbers for those under 1 year and those under 5 years, with a male predominance under 1 year and a female predominance under 5 years. Presenting conditions followed South African trends^30^ of causes of death in those under 5 years with pneumonia first, followed by seizures and diarrhoeal diseases.

### Caregivers’ experiences

It is not surprising that 74% of survey respondents experienced barriers to accessing care, given what is known from national and international literature.^3,4,8,9,17,18,31^ The literature shows that foreign nationals experience greater barriers concerning language, staff attitudes, transport, xenophobia and documentation.^8,31^ The former three featured prominently in interviews and surveys, while some survey respondents felt discriminated against because of their ethnicity.

Barriers to access elicited by the survey were similar to those expressed in the interviews. The barriers experienced were related to the associated cost of health care (affordability), transport costs and loss of income for days spent at the hospital being the dominant concerns. Many found the waiting times and staff attitudes unacceptable. Accessibility was influenced by language barriers, distance to a health care facility, the safety of the area, inadequate capacity of the facility, and lack of ambulance services. One interview highlighted the difficulty caregivers face in accessing care after-hours in an area serviced only weekly by a satellite clinic (availability). Household barriers evident in both the interviews and survey were mostly related to inadequate knowledge of treating the illness at home, recognising the severity of the illness and practices such as washing the child’s mouth with soap after poison ingestion. Many would not initiate SSS at home for diarrhoea or vomiting, and some would wait for signs of respiratory distress before taking a child with a respiratory illness to the hospital. Many did not know the EMS number.

The coronavirus pandemic impacted healthcare attendance. Child primary health care visits were reduced by up to 60% in some areas of the country.^32^ Local data from another subdistrict in Cape Town showed a reduction in child emergency presentations.^33^ Those who did present were generally more ill compared with pre-pandemic presentations,^33^ which may indicate a delay in health-seeking behaviour.

### Providers’ ideas on improving access to care for children

The findings of the NGT conducted with providers concur well with those of the caregivers’ experiences. Understanding of caregivers’ experiences is important in devising solutions proposed by providers. The top-ranked items from the combined list generated by participants in (stages 2–4) involved improving ambulance services, extending service opening times (to 21 h or 24 h) at the local clinic and including ambulance and emergency telephone numbers in the RTHB. Solutions proposed by the nominal group will be relayed to the district paediatrician to be addressed by future research for example piloting interventions. Providers noted the role of outside stakeholder solutions, for example, by higher management, such as improved government funding for PHC, transport vouchers, ambulance availability and adjustment of clinic service hours.

### Recommendations

Recommendations have been categorised into the themes of barriers experienced; however, some recommendations will affect multiple barriers. These recommendations are a combination of the recommendations from the NGT and the authors’ recommendations. See Box 1 for a summary of the recommendations:

- Affordability could be addressed through transport vouchers or improving private–public partnerships. The utility of this community asset has been illustrated in Masiphumelele with private ambulances assisting state patients. Lost income for days spent at the clinic may be alleviated if existing appointment systems are improved, opening hours extended and families seen by the same doctor rather than at separate appointments. The implementation of the proposed National Health Insurance (NHI) may narrow the access gap between the insured and uninsured.
- Acceptability

BOX 1 Summary of recommendations.

**Summary of recommendations**

1. **Affordability**
  - Transport vouchers
  - NHI
  - Appointments and longer clinic hours
2. **Acceptability**
  - Staff training
  - Appointments
3. **Access**
  - Crime reduction
  - Translation services
  - Transport solutions
  - Improved social capital
  - Increased PHC spend.
4. **Availability**
  - Clinic hours adjustment
5. **Household factors and facility factors**
  - CHWs
  - Education
  - Publication – 112
  - Increase scope of CHWs
  - Increase CHW workforce

PHC, primary health care; CHW, community health workers; NHI, National Health Insurance.

Acceptability of the healthcare service may be enhanced by staff training in interpersonal communication, compassion^34^ and the sensitive handling of patients and family members. It is worth investigating the achievability in our area. All staff should be trained in this regard. Family friendly methods of accessing care are sought, for example, family-orientated clinics and/or clinics available after hours for working care givers.

- Access and availability

A community-based ambulance service would be a facilitator to access by expansion of the Emergency First Aid Responder^35^ (EFAR) programme. Emergency First Aid Responder is a programme that trains community members in basic first aid with the idea that they can be called upon as the first responders to an emergency within the community and then co-ordinate with EMS. Emergency First Aid Responders could assist with basic home care, directing the ambulance to the location when called and to ascertain if an ambulance is really warranted. Strategies to address language barriers such as on-site translators or 24-h access to the Folio translation service are needed. High crime rates require community-wide interventions. False Bay Hospital is the only 24-h facility in the Far South. Extended opening hours at local PHC services will improve the availability of services and may overcome some transport barriers.

- Household and community factors

Community health workers should focus on health promotion for appropriate health-seeking behaviours. Profile mapping can focus resources on high-risk families. Health education at multiple levels is needed including community health promotion, education at primary and high schools, using social media, radio and television; and educating pregnant women and parents to recognise when a child is sick and what home care can be provided this is termed health literacy.^36^ Community-based services (CBS) should be strengthened and the ratio of CHWs to households should be increased,^12,15,37^ thus enriching lives through the COPC approach. This model has been met with success at Philani,^38^ a local NGO active in the Western Cape for more than 40 years. The training of community-based workers under different NGOs is often disparate thus standardisation of the CHW curriculum is needed.^39^

It is recommended that the Maternal, Women, Child, Adolescent and Neonate (MWCAN) forum gives as much attention to respiratory illnesses in children now as it did with diarrhoeal diseases over the past decade. This is a metro-wide clinical governance forum that deals with both paediatric, adolescent, maternal and women’s health.

The authors observed inaccurate data capturing of dead-on-arrival children. This highlights the need for a standard operating procedure across all the services. At the time of the study, FBH used a paper-based register system. Advances in technology such as Hospital and Emergency Centre Tracking Information System (HECTIS) – an electronic triage and emergency record-keeping for emergency centres – have the potential to improve data capturing and reduce triage errors.

The study’s findings should lead to policy considerations that could help staff relate to the patients and handle difficult situations. Further research into implementing solutions is needed. This could focus on reducing out-of-hospital deaths through improved CBS. These measures are encouraged by the first 1000-day programme and COPC and PHC policies. The PHC budget needs to be increased and studies should examine whether there is a relationship between child mortality and the number of homes each CHW visits.

Technologies including artificial intelligence could be studied to determine more effective ways of utilising current resources. This could use mapping the geographic relationships of communities and healthcare facilities and the most efficient transportation routes between them, modelled on previous encounters. Having an ambulance base in the far south could influence ambulance availability. A 24-h community health centre (CHC) within the Far South is important for improving access to care. Although not addressed by our study, it is likely that providers have lower confidence in handling paediatric emergencies because of seeing them infrequently (average two per month), which is consistent with a recent publication by Amien.^40^ Confidence in handling emergencies could be addressed with regular simulation training.

Local assets and facilitators suggested in this study should be investigated further to reduce mortality in line with the provincial health plan.^27^

### Limitations

The study faced several constraints. A potential limitation was the interpretation of data represented in other languages. However, translators were used to ensure the integrity of the data.

Not all registers or files were available and triaging errors excluded some potential interviewees. Not all potential interviewees were contactable; this could introduce bias as those who are uncontactable may experience more barriers to accessing care. Interviews were conducted by a trained research assistant with counselling experience; however, some interviews were more structured and some more semi-structured reflecting educational and language difficulties in the interviews. This was not felt to detract from the data quality and did not impact saturation of themes. A potential bias of qualitative data collection and representation is the subjective views of the researcher being incorporated into data through suggestive questioning or selective quotation. This was mitigated by the electronic recording of all responses transcribed verbatim. All transcripts were reviewed by multiple researchers and comparisons were made to confirm themes represented to ensure credible, dependable and reliable representation of the data. Member checking was not performed and those interviewed were not contacted again to confirm what they said or if they were happy with the transcripts. Not all demographic data of the interviewees were recorded consistently. The presence of a representative from EMSs and a nursing sister in charge of Masiphumelele clinic in NGT would have strengthened the results. These key stakeholders will be engaged in implementing recommendations. As a result of restrictions imposed during the coronavirus pandemic, in-person voting, and prioritisation (stage 5) did not take place at a follow-up meeting. These were replaced by ranking and voting by email and did not include the whole group – a further limitation. Despite the constraints forcing a modification of the NGT, the generation of ideas (stages 3 and 4) in response to the question posed was ensured.

The constraints notwithstanding, a strength of the study is the inclusion of caregivers’, community and providers’ voices that also permitted triangulation and integration of data.

## Conclusion

The study’s findings are based on the experiences of users and providers of child health services and concur with other studies locally and internationally. The barriers highlighted in this study are likely to reflect those in the rest of South Africa. The study results suggest an association between suboptimal caregiver care at home for sick children combined with barriers to healthcare access and the higher childhood mortality in the out-of-hospital group in this subdistrict. The facilitators to healthcare access identified by the NGT propose a strong voice for change.

A set of recommendations were made that could inform health service planning and implementation. Further research should focus on community-based interventions that improve health-seeking behaviour and explore early warning systems for the child-at-risk.
